# One Health Approach to Tackle Microbial Contamination on Poultries—A Systematic Review

**DOI:** 10.3390/toxics11040374

**Published:** 2023-04-14

**Authors:** Bianca Gomes, Marta Dias, Renata Cervantes, Pedro Pena, Joana Santos, Marta Vasconcelos Pinto, Carla Viegas

**Affiliations:** 1 CE3C—Center for Ecology, Evolution and Environmental Change, Faculdade de Ciências, Universidade de Lisboa, 1749-016 Lisbon, Portugal; 2 H&TRC—Health & Technology Research Center, ESTeSL—Escola Superior de Tecnologia e Saúde, Instituto Politécnico de Lisboa, 1990-096 Lisbon, Portugal; 3 NOVA National School of Public Health, Public Health Research Centre, Comprehensive Health Research Center, CHRC, NOVA University Lisbon, 1600-560 Lisbon, Portugal; 4 CISAS—Center for Research and Development in Agrifood Systems and Sustainability, Instituto Politécnico de Viana do Castelo, 4900-347 Viana do Castelo, Portugal; 5 Polytechnic Institute of Coimbra, Escola Superior de Tecnologia da Saúde de Coimbra, Rua 5 de Outubro, 3046-854 Coimbra, Portugal

**Keywords:** One Health approach, exposure assessment, microbial contamination, poultries, food safety

## Abstract

This study reports the search of available data published regarding microbial occupational exposure assessment in poultries, following the PRISMA methodology. Air collection through filtration was the most frequently used. The most commonly used passive sampling method was material collection such as dust, cages, soils, sediment, and wastewater. Regarding assays applied, the majority of studies comprised culture-based methods, but molecular tools were also frequently used. Screening for antimicrobial susceptibility was performed only for bacteria; cytotoxicity, virological and serological assays were also performed. Most of the selected studies focused on bacteria, although fungi, endotoxins, and β-glucans were also assessed. The only study concerning fungi and mycotoxins reported the carcinogenic mycotoxin AFB1. This study gives a comprehensive overview of microbial contamination in the poultry industry, emphasizing this setting as a potential reservoir of microbial pathogens threatening human, animal, and environmental health. Additionally, this research helps to provide a sampling and analysis protocol proposal to evaluate the microbiological contamination in these facilities. Few articles were found reporting fungal contamination in poultry farms worldwide. In addition, information concerning fungal resistance profile and mycotoxin contamination remain scarce. Overall, a One Health approach should be incorporated in exposure assessments and the knowledge gaps identified in this paper should be addressed in further research.

## 1. Introduction

The One Health approach incorporates human, animal, and plant health, as well as the health of their shared environment, for supporting a multidisciplinary and holistic approach that integrates monitoring, planning, and evaluation to optimize co-benefits and public health outcomes [1,2]. In addition, the One Health approach supports global health by fostering coordination, collaboration, and communication among different sectors at the human–animal–environment interface to address common health threats such as antimicrobial resistance (AMR), food safety, zoonotic diseases, and several others [2,3].

The industrialization of the poultry sector poses a considerable negative impact on air, soil, and water. The increase in waste management problems can be considered as one of the major drivers fostering harmful effects on environmental health [4]. Indeed, pathogens can be disseminated by unrecognized pathways, for example, on airborne dust and animal wastes utilized in agriculture and, consequently, water and soil quality may be impacted [5].

Poultry production intensification needs increases in livestock numbers and densities, the use of particular feed to raise conversion ratios, and shorter production cycles [4,6]. Consequently, such changes may potentially alter transmission patterns and the evolutionary conditions of dominant pathogens, leading to emergence of zoonotic diseases [4,7]. The environment of animal husbandry, such as humidity level, number of animals, ventilation type, and hygiene measures may influence microbial development [8]. In fact, intensive animal production is also considered as one of the causes for biodiversity loss and potentially for upcoming pandemics [2,9].

Agricultural expansion and intensification bring wildlife, livestock, and people into closer contact, allowing animal microbes to spill over into people and causing infections, sometimes outbreaks, and less frequently epidemics and pandemics [1,2]. Production intensification of livestock raises concerns about the feasibility of the One Health model for animal production regarding the protection of the health of animals, workers, and consumers [10]. Thus, intensive poultry farming not only poses a significant risk to workers [11,12] but can also act as a potential public health menace [1,4].

Human and animal well-being is also in the scope of a One Health approach. Animal diseases threaten human health, food safety, and security, driven by the transmission of zoonotic diseases or by the loss of animal productivity. Adequate hygiene management is therefore critical to avoiding the negative human health and economic repercussions of foodborne diseases [13].

We shouldn´t consider the close linkage and interdependencies of human and animal health without considering maintenance of stable ecosystem services that can be threatened by livestock rearing methods and/or excessive exploitative human activities [14]. In 2013, the European Union (EU) through the Directive 2003/99/EC aimed to improve the system for monitoring and collection of information on zoonoses, antimicrobial resistance, and foodborne outbreaks (EU, 2013). In 2017, the European Centre for Disease Prevention and Control (ECDC), European Food Safety Authority (EFSA), and European Medicines Agency (EMA) jointly established a list of harmonized outcome indicators to assist EU Member States’ assessment of the progress in reducing the use of antimicrobials and AMR in both humans and food-producing animals (ECDC, EFSA, EMA; 2017). More recently, in 2021, a list of harmonized outcome indicators was presented per country in the scope of the One Health approach (ECDC, EFSA, EMA; 2021). Additionally, since 2011, EFSA has reported zoonoses, zoonotic agents, and foodborne outbreaks and, in 2019, the annual EU Summary Reports were renamed the “EU One Health Zoonoses Summary Report” which is co-authored by EFSA and ECDC. In the scope of food safety, the poultry industry remains a public health issue, since foodborne pathogens can be in contact at all phases of the producing chain. Thus, identifying the sources and routes of transmission of pathogens is required in order to reduce their occurrence. Some regulatory guidelines have been designed to answer these concerns, namely the Regulation (EC) No 2073/2005, concerning microbiological contaminants for food safety criteria. However, the food industry is known to be very committed to food safety assurance, but less concerned with the safety of workers, and biological risk assessment is usually neglected by occupational health professionals due to the lack of systematized information about the biological agents involved [15]. In fact, the microorganisms’ occupational exposure is being neglected in a wide range of industrial sectors (besides poultry production), being less recognized and not so well described in comparison with other occupational agents [16].

Concerning the occupational health legal framework, Portuguese employers are obliged by regulation to assess and prevent exposure to occupational risks [17] and specifically to biological agents. The Directive 2000/54/EC of the European Parliament and the Council of September 18 sets the rules regarding risk assessment if exposure to biological agents cannot be avoided [18]. However, it is not common to include zoonosis and AMR as a source of risk in studies on occupational risk assessment of animal-related occupations. In 2020, the European Agency for Safety and Health at Work (EU-OSHA) published a review about biological agents and prevention of work-related diseases, and animal farming was considered a high-risk occupation [19].

Due to the lack of studies regarding poultry farms this study aimed to perform a systematic review to provide a broad overview of the state of the art in the developed subject, describing the microbiological contamination reported in previous studies developed in poultry farms and indicating which parameters and methods were applied to perform the microbial contamination assessment in this setting in different scopes (occupational/food safety/animal health).

## 2. Materials and Methods

### 2.1. Registration

The Preferred Reporting Items for Systematic Reviews and Meta-Analyses (PRISMA) checklist [20] was completed (Supplementary Material—Table S1).

### 2.2. Search Strategy, Inclusion and Exclusion Criteria

This study reports the search of available data published in the period of 1 January 2000 to 20 January 2023. The search terms aimed to identify studies on microbial occupational exposure assessments, selecting studies on sawmills that included the terms “exposure” AND “microorganisms” AND (“poultry” OR “broilers”), with English as the chosen language. The databases chosen were PubMed (https://pubmed.ncbi.nlm.nih.gov/ (accessed on 20 January 2023)), Scopus (https://www.scopus.com/ (accessed on 20 January 2023)), and Web of Science (WoS) (www.webofscience.com (accessed on 20 January 2023) following the PRISMA methodology. This search strategy identified 258 papers in all databases. Articles that did not fulfill the inclusion criteria were not subjected to additional review (but some of them were used for Introduction and Discussion sections) (Table 1).

### 2.3. Study Selection and Data Extraction

The selection of the articles was performed through the Rayyan intelligent systematic review application, which is a free web-tool that greatly speeds up the process of screening and selecting papers for academics working on systematic reviews, in three rounds.

The first round was conducted by one investigator (BG) and comprised the screening of all titles to eliminate papers that were duplicated or unrelated to the subject. 

Rayyan was then used to analyze the papers that were chosen. The second round was a screening of all abstracts carried out by two investigators (BG and RC). The full texts of all potentially relevant studies were evaluated in the third round, taking into account the inclusion and exclusion criteria. Potential divergences in the selection of the studies were analyzed and resolved by four investigators (BG, MD, RC, and PP). Data extraction was then conducted by BG. It was also checked over by MD and CV. The following details were manually extracted: (1) databases, (2) title, (3) country, (4) environment assessed, (5) objective, (6) microorganisms and metabolites, (7) analyzed matrices, (8) sampling methods, (9) analytical methods, (10) main findings, (11) references.

### 2.4. Quality Assessment

The assessment of the risk of bias was performed by two investigators (BG and CV). Within each study, we evaluated the risk of bias across three parameters divided into key criteria (environment assessed, microorganisms and metabolites, sampling methods, analytical methods). 

Each parameter’s risk of bias was rated as “low,” “medium,” “high,” or “not applicable.” The studies for which all the key criteria and most of the other criteria were characterized as “high” were excluded.

## 3. Results

**Figure 1:**
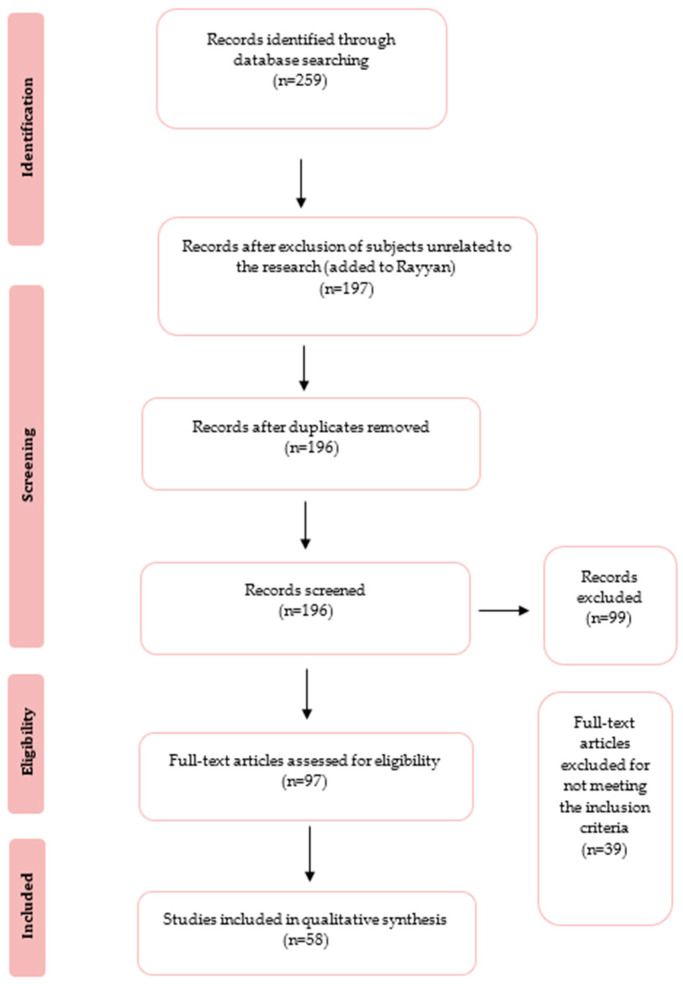
PRISMA-based selection of articles.

The workflow diagram for selecting studies is illustrated in Figure 1. Initially, 259 studies were found in the database search, from which 197 abstracts were examined and 97 complete texts were assessed for eligibility. After considering the inclusion and exclusion criteria, a total of 39 studies were disregarded, mostly because they were related to biocontrol efficacy, clinical trials, or biological samples. A total of 58 studies related to microbial exposure in poultry facilities were selected.

### Characteristics of the Selected Studies

Table 2 describes the main characteristics of the selected studies. Of the reviewed studies (n = 58), 34 were conducted in Europe (9 in Germany [21,22,23,24,25,26,27,28,29], 8 in Poland [25,30,31,32,33,34,35,36], 4 in Portugal [37,38,39,40], 3 in Italy [41,42,43], 1 in the Netherlands [44], Spain [45], Austria [46], Lithuania [47], France [48], Denmark [49], 1 in Denmark and Switzerland [50], and 2 in several European countries (Belgium, Bulgaria, Denmark, France, Germany, Italy, the Netherlands, Poland, and Spain [51] and Denmark, Switzerland, and Spain [52]). Thirteen studies were performed in Asia, namely, six in China [39,53,54,55,56,57], four in Korea [58,59,60,61], one in Lebanon [62], one in India [37], and one in Iran [63]. Eight studies were carried out in America, including six in the United States of America [64,65,66,67,68,69] and two in Canada [70,71]. In Africa, three studies were conducted in Egypt [72,73,74] while, in Oceania, two studies were performed in Australia [75,76].

.

The majority of studies (31 out of 58, 53%) were performed on poultry farms [23,24,27,28,30,31,32,35,36,38,40,41,43,44,48,49,54,55,57,60,62,63,66,68,69,70,71,72,74,76,78]; followed by animal farms (19 out of 58%, 33%), namely, six on poultry and swine farms [40,50,51,52,58,64], two on poultry and dairy farms [37,53], two on poultry farms, sheep sheds, and horse stables [33,49], one on cattle, chicken, swine, and sheep [67], one on poultry, swine, and cattle [59], one on cow barns, swine, sheep sheds, poultry houses, horse stables, and buildings for storage of hay [34], one on poultry, pork, and beef farms [45], one on a duck hatchery [22], one on a turkey hatchery [21], one on swine, chicken, and cattle farms [61], one on poultry farms and live poultry markets [56], and one on poultry processing plants [75]. Additionally, three studies (5%) covered several workplaces (one on a cement plant, composting plant, poultry farm, and cultivated area [25], one on a poultry farm, flourmill, and the textile and food industry [73], and one on a poultry farm, swinery, feed preparing and storing house, grain mill, wooden panel-producing factory, and organic waste and recycling facilities [47].

Most of the studies focus on occupational health (33 out of 58, 57%) [21,22,23,24,25,26,27,28,30,31,36,38,39,40,41,42,46,47,48,49,50,51,52,55,58,59,60,64,66,67,70,73,74], followed by food safety (6 out of 58, 10%) [43,45,49,69,71,75] and human health (5 out of 58, 9%) [32,53,56,63,77]. Some studies encompass occupational and animal health (4 out of 58, 7%) [33,35,54,61], while some studies focus only on animal health (4 out of 58, 7%) [44,57,68,76]. Few studies focused on human and animal health (2 out of 58, 3%) [78]. One study focused on environmental health and food safety [29], one on human and environmental health [62], and one on environmental health [37].

Regarding microbial contamination, most of the selected studies focused on bacteria (31 out 58, 53%) [21,22,23,24,26,27,28,29,30,35,37,43,44,45,49,51,53,54,55,56,57,62,63,64,67,68,69,71,75,76,77], while some considered bacteria and fungi (8 out 59, 14%) [25,46,49,58,60,66,73,74]. Eight studies (14%) considered microorganisms and metabolites, six studies (10%) focused on bacteria, fungi, and endotoxins [42,50,52,59,61,72], one focused on bacteria, fungi, endotoxins, and β-glucans [31], and one focused on bacteria and endotoxins [70]. In addition, six studies (10%) focused only on fungi [32,36,38,39,47,78], three studies (5%) on endotoxins [33,34,48], one study on fungi and mycotoxins [40], and one study on viruses [41]. 

The most frequent sampling method was air sampling (36 out of 58, 62%) [21,22,23,24,26,27,28,30,31,32,34,35,36,38,39,42,46,47,48,50,52,53,54,55,57,58,59,60,61,64,65,66,70,73,74,78]. Considering active sampling, air collection through a filtration method was frequent (24 out of 58, 41%) [21,22,23,24,26,27,28,30,31,34,36,42,47,48,49,50,52,55,57,59,61,64,70,73]. The impaction method was also recurrent (12 out of 58, 21%) [31,32,38,39,46,54,58,59,60,61,70,78], followed by the impingement method (4 out of 58, 7%) [23,46,47,73]. Additionally, one study used the two-stage bioaerosol cyclone [27].

Regarding passive sampling, material collection was the most frequent methodology applied (13 out of 58, 22%) [21,33,34,35,37,38,45,51,56,62,68,69,71], namely of dust (3 out of 58, 5%) [33,35,51], litter samples (3 out of 59, 5%) [38,62,68], bird carcasses (3 out of 58, 5%) [45,69,71], and animal fecal samples (2 out of 59, 3%) [69,71]. Manure and soil samples [37], eggshell [21] and specimens, feces, cages, soils, sediment, wastewater, and surface swabs from chopping boards [56] were collected. Some studies performed surface swabs (11 out of 58, 19%) [22,39,41,43,44,49,67,71,74,76,77] of bird cloacas (5 out of 58, 8%) [43,44,49,71,77], bird cloacas and human sera [41], human sera [22], the skin of workers [67], nose and throat of workers [74], fecal droppings [76], and the floor [39]. Furthermore, three studies used both active and passive sampling methods [39,40,74] and two studies performed personal air sampling [50,74].

Considering analytical procedures for microbial characterization, the majority of studies used culture-based methods (41 out of 58, 71%) [21,23,24,25,28,29,30,31,32,35,36,37,38,39,40,42,43,44,45,46,47,49,52,53,55,58,59,60,61,62,63,65,68,69,71,72,73,74,75,76,77]. Fluorescence quantification was also recurrent (5 out of 58, 9%) [21,22,27,50,52]. In addition, screening for antimicrobial susceptibility was carried out only for bacteria (13 out of 58 studies, 22%) [29,37,43,45,49,53,63,65,69,71,72,75,77]. In addition, two studies performed a cytotoxicity assay [25,35] and one study used both virological and serological assays [41].

Molecular tools were frequently applied (29 out of 58 studies, 50%) [21,22,23,24,25,26,27,28,29,37,38,39,43,44,49,51,53,54,56,57,64,65,66,67,70,71,75,77,78]. Several studies performed PCR (9 out of 58, 16%) [23,24,25,38,39,54,57,71,77], while some resorted to RT-PCR [43,53]. Antibiotic resistance gene sequencing was carried out by four studies (7%). While some studies rely on metagenomics analysis (2 out of 58, 3%) [51,56], others rely on whole-genome sequencing [29], while one study used restriction fragment length polymorphism [27] and other pyrosequencing analyses [66]. Several studies performed gene sequencing (20 out of 58, 33%), with the 16s rRNA gene being the most frequently sequenced (11 out of 58, 19%) [21,22,23,26,27,28,44,53,57,64,67], followed by vanA PCR (2 out of 58, 3%) [49,65]. Additionally, one study performed 16s rRNA, tetL, tetW, and *E. coli* gene sequencing [54], and one study sequenced tetracycline resistance genes [35]. Gas chromatographic and spectrophotometric methods were also used by some studies (5 out of 58, 9%) [33,34,35,70,72]. 

Regarding metabolite characterization, Limulus amebocyte assay was frequently used for endotoxin assessment (11 out of 58, 19%), while one study used ELISA assay for mycotoxin assessment [40]. On the other hand, chemical analysis of litter samples was performed by one study [62]. Furthermore, quantitative kinetic Glucatell assay was used for β-glucan assessment [31]. Additionally, some studies performed questionnaires [50,74] and spirometric measures from workers were performed [50,74] .

According to the microbial assessment, several studies evidence the presence of bacteria (26 out of 58, 45%) [21,22,23,25,27,30,35,43,44,51,53,54,55,60,61,62,64,68,69,70,71,72,73,74,75,77] in facilities associated with poultry production. Fungal exposure was also evidenced by 14 studies (24%) [25,32,35,36,38,39,42,60,65,66,72,73,74,78]. Furthermore, microorganisms´ metabolites such as endotoxins were detected by eight studies (14%) [33,42,48,53,59,61,70,72], while endotoxins and β-glucans were evidenced by one study [31]. The only study that performed fungal and mycotoxin assessment found the carcinogenic mycotoxin AFB1 [40]. Overall, occupational exposure to microorganisms is a frequent concern reported by the selected studies (15 out of 58, 26%) [22,23,27,31,33,35,36,40,41,42,48,55,67,70,72]. Indeed, the risk of exposure to potential pathogenic bioaerosols originating in poultry facilities is emphasized in some studies (5 out of 58, 9%) [21,54,55,59,60].

## 4. Discussion

Industrialization has led to increased animal density in enclosed production buildings, resulting in high concentrations of viable and non-viable bacteria and fungi, as well as metabolites in bioaerosols [21]. The poultry industry has been found to pose a significant global health risk due to microbiological contamination [73]. Farm facilities housing multiple animals promote complex mixtures of microorganisms in bioaerosols, including dust-containing feathers, skin fragments, feces, feed particles, microorganisms, and chemicals [74]. Long shifts in manufacturing plants have become common, resulting in workers inhaling complex bioaerosols, which can pose several health hazards in agricultural environments [21]. This situation has prompted increased studies on occupational health. Bioaerosols from farms can also pose health risks to nearby residents [53,74], highlighting the importance of research on human health, environmental impact, and the One Health approach to address these concerns. Broilers and laying hens are susceptible to bacterial and viral infections of the upper respiratory tract, as indicated by several studies [38,51,62,70]. The transmission of pathogens can occur through inhalation, close contact with infected animals, feces, litter, or contaminated objects, and inadequate biosecurity controls can result in significant economic losses [74]. As international trade expands, food safety concerns regarding the rapid spread of foodborne pathogens through the global food chain are increasing [73].

Moreover, environmental health concerns arise from the utilization of animal by-products, such as poultry manure and litter, in agriculture. Repeated use of these by-products as manure can lead to the accumulation of contaminants in agricultural soils, potentially increasing their bioavailability and toxicity in the environment [74]. Air sampling has been widely used to characterize occupational exposure to fungi, but it is important to consider the appropriate sampling period and the influence of variables such as ventilation and building features. Passive sampling methods, such as settled dust assessment, have been shown to be more reliable for collecting contamination over a longer period of time. Broiler manure and animal bedding have been identified as the primary sources of indoor air microbial contamination in the poultry industry [76,77,78,79,80,81,82,83].

It is recommended to use a multiapproach sampling protocol for a more comprehensive understanding of microbial contamination. While culture-based methods have been primarily used for microbial characterization, culture-independent methods such as cloning approaches and quantitative real-time PCR have shown to be suitable for various bioaerosol measurements. Molecular tools, such as whole-genome sequencing, could provide more information on the biodiversity of microorganisms in these environments. Overall, these findings highlight the importance of considering various sampling methods and assays in the assessment of indoor microbial contamination in the poultry industry [60,61]. Studies on bioaerosols in poultry production are limited and identifying all organisms, both viable and non-culturable, is important for characterizing bioaerosols in these facilities [60]. Inhalation exposure to non-viable microorganism components such as endotoxins and mycotoxins may cause health hazards, so evaluating non-viable components may be useful for assessing pulmonary disease risk. Microbial assessment of poultry farms shows the presence of numerous microbes, including zoonotic pathogens, which can act as transport agents of airborne diseases [49,61]. Despite the growing threat of fungal infections to human health, there are fewer studies conducted on fungi (and also viruses) compared to bacteria, and this lack of attention and resources makes it challenging to determine the precise burden of fungal infections and to encourage policy and programmatic action [75].

Due to the extensive use of antibiotics in the livestock industry, these facilities are significant sources of antibiotic resistance genes (ARGs). Therefore, multidrug-resistant bacterial pathogens may be transmitted through the inhalation of bioaerosols [55]. This explains the frequent screening for bacterial antimicrobial susceptibility by several studies [29,37,43,45,49,53,63,69,71,72,75,77]. 

Several potentially pathogenic bacteria have been identified [24,30,75,77]. The potential dispersal pattern and distance of airborne bacteria and ARGs from these animal sources remain unknown [53]. However, it is important to note that clinically significant multidrug-resistant bacteria *Staphylococcus* sp. [53,75], *E. coli* [29], *Campylobacter jejuni* [71], among others, belonging to the WHO priority pathogens list of antibiotic-resistant bacteria (2017), were isolated from poultry farms. 

Recently, the World Health Organization (WHO) published the first fungal priority pathogens list [79], listing 19 groups of human fungal pathogens associated with a high risk of mortality or morbidity. This formal recognition by the WHO highlights an important group of infections, which has been perennially neglected in terms of the awareness and research funding needed [80]. 

Regarding fungal assessment, despite the low number of studies (14 out of 58, 24%), several fungi comprising the critical priority group of the WHO list (2022), namely, *A. fumigatus,* were frequent in indoor air samples [32,36,47], along with *Candida albicans* [32]. Regarding the high-priority group, *Fusarium* sp. [32,38,47], the order Mucorales [40,47], and *Candida tropicalis* [36] were also some of the ones reported.

Concerning microbial components, endotoxin, a major component of the outer membrane of Gram-negative bacteria, poses a serious health risk [34]. Endotoxins found in airborne organic dust have been linked to respiratory disease in both humans and animals [34].

Regarding mycotoxins, some of the literature already evidenced occupational exposure in animal production facilities [40]. In fact, fungal species recognized as mycotoxin producers were reported in some of the selected studies [32,36,42,78]. Even though only one of the selected studies performed mycotoxin assessment, the obtained results are enough to hypothesize that workers in these settings may be at a higher risk of *Aspergillus* mycotoxicosis. Indeed, elevated concentrations of *A*. *flavus* and *A. versicolor* were recovered through environmental sampling. Additionally, through human biomonitoring, analysis of mycotoxins and/or their metabolites in blood and urine evidence detectable levels of the carcinogenic mycotoxin AFB1 [40].

Briefly, to mitigate and decrease such pollutants it is crucial to establish international standards for what constitutes good microbiological indicators from environmental samples, which could be used to guide risk reduction decisions and create effective incentives for people to follow such guidance, which have already been suggested [81].

Globally, temperature rises due to climate change have various impacts on ecosystems, human health, animal health, and food production, which also affect AMR [81]. 

The emergence of resistant fungal strains in occupational exposure scenarios has already been demonstrated [82,83]. Indeed, temperature increases may influence the susceptibility of pathogens (bacteria, fungi, and parasites) in chicken environments [84]. Thus, as in the case of bacteria, antifungal resistance should be addressed in further research [85,86]. Additionally, it is crucial to investigate the effects of heat stress on poultry production to formulate various effective mitigation strategies to reduce significant production losses [84].

The prevalent airborne microorganisms in animal production buildings are not well characterized in terms of quantity, composition, and risk group. Identification and quantification would be useful for determining the causative agents and performing risk assessments [27].

The poultry industry must be sustainable, and it needs to produce more with less, while benefiting all [87]. The sector must improve human, animal, and environmental health and welfare. Implementing a comprehensive and coordinated One Health approach that incorporates exposure assessment can help tackle threats to health and ecosystems [81], ensuring priority areas for action in order to mitigate microbial exposure, promoting a safe environment for workers and animals in poultry facilities, along with less environmental impact.

Overall, these findings highlight the need for improved biosecurity measures and environmental management practices to ensure animal health, food safety, and environmental sustainability in the poultry industry.

## 5. Conclusions

This review allowed us to identify microbiological contamination reported in the poultry industry, sampling methods and assays already employed to assess occupational exposure to microbial contamination within different scopes (occupational health, food safety, and animal health), and knowledge gaps to be tackled in future studies. 

Poultry workers are exposed to several microbial contaminants in their workplace. Exposure to bacteria and fungi has been assessed and reported, as well as bacterial metabolites (namely endotoxins), β-glucans, and mycotoxins. Occupational exposure to microorganisms is a frequent concern, and the risk of exposure to potential pathogenic and resistant bioaerosols originating in poultry facilities is emphasized. Future research should aim to identify the main sources of contamination in this setting.

A One Health approach is a vital framework and the use of effective risk assessment tools and strategies can help prevent occupational exposure and protect the health of workers, consumers, and animals. 

## Figures and Tables

**Table 1:** Inclusion and exclusion criteria for the articles selected.

Inclusion Criteria	Exclusion Criteria
Articles published in the English language	Articles published in other languages
Articles published from 1 January 2000 to 20 January 2023	Articles published prior to 2000
Articles reporting findings from any country	Articles related to biocontrol efficacy or related to clinical trials
Articles related to microbial exposure from poultries and related products	Articles related to biocontrol efficacy or without mention of microbial exposure or metabolites
Original scientific articles on the topic	Abstracts of congresses, reports, reviews/state of the art articles

**Table 2:** Characteristics of and Data Obtained in the Selected Studies.

Database	Title	Country	Environment Assessed	Objective (Occupational/Food Safety/Public Health/Animal Health)	Microorganisms and Metabolites	Analyzed Matrices	Sampling Methods	Analytical Methods	Main Findings	References
PubMed	Occupational exposure to aflatoxin (AFB₁) in poultry production	Portugal	Poultry farm	Occupational health	Fungi Mycotoxins	Air samples Surface swabs Litter collection (poultry) Floor coverage collection (swine) Workers’ biological samples (blood: poultry farms n = 31)	Active methods Impaction flow rate = 140 L/min) Passive methods (material collection; swabs)	Culture-based methods ELISA	Eighteen poultry workers (58.6%) and six workers from the swine production facilities (54.5%) showed detectable levels of aflatoxin B1 (AFB1). The findings indicate that AFB1 inhalation exposure occurs in both occupational settings, posing an additional risk that must be identified, assessed, and avoided.	[40]
	Bioaerosol exposure by farm type in Korea	Korea	Animal farms (open field, greenhouse and livestock facilities: poultry, swine, and cattle)	Occupational health	Bacteria Fungi Endotoxins	Air samples (open field farms: personal n = 4, environment n = 20) Greenhouses: personal n = 32, environment n = 159 Livestock facilities: environment n = 21, poultry n = 9; swine n = 5; cattle n = 5)	Active methods (single-stage impactor, flow rate = 28.3 L/min; button aerosol sampler with sterilized gelatin filters, flow rate = 4 L/min; two-stage cassette with a glass fiber filter for endotoxins, flow rate = 2 L/min)	Culture-based methods Limulus amoebocyte lysate (LAL) assay (endotoxins)	The highest endotoxin concentration was at hog farms (160.35 EU/m^3^), followed by poultry houses (103 EU/m^3^) and cowsheds (28 EU/m^3^). The measured levels of endotoxins at hog farms and poultry houses exceeded exposure limits. The concentrations of personal samples were higher than those of the area samples. Exposure levels in residential and rest areas were significantly higher than in the control areas, possibly being contaminated from bioaerosols generated in the workplace.	[59]
	Serologic Evidence of Occupational Exposure to Avian Influenza Viruses at the Wildfowl/Poultry/Human Interface	Italy	Poultry farm (n = 17)	Occupational health	Viruses (avian influenza viruses)	Bird cloacal swabs (n = 2542) Oropharyngeal swabs (n = 1045) Avian sera (n = 2688) Human sera (n = 57 workers) and blood samples	Passive methods (swabs, material collection) Biological samples	Virological and serological assays(hemagglutination inhibition assay; enzyme-linked immunosorbent assay (ELISA))	Antibodies specific to avian influenza viruses (AIH)-H3, AIV-H6, AIV-H8, and AIV-H9 were found in three poultry workers (PWs). The data obtained emphasize the occupational risk posed by zoonotic AIV strains. These findings highlight the crucial role of integrated occupational medicine and veterinary avian influenza virus surveillance aimed to further assess the health risk at the wildfowl/poultry/human interface	[41]
	Spatiotemporal variations in the association between particulate matter and airborne bacteria based on the size-resolved respiratory tract deposition in concentrated layer feeding operations	China	Poultry farms (n = 9)	Occupational health	Bacteria	Air samples (n = 8)	Active methods (Andersen eight-stage samplers, n = 2; Andersen six-stage samplers, n = 2, flow rate = 1cubic foot/min) Particulate matter (PM) collected on the surface of a glass fiber filter membrane with a diameter of 81 mm and pore size of 2.0 μm)	Culture-based methods	The emissions of PM and airborne bacteria (AB) from the poultry houses resulted in high PM and AB concentrations in the surrounding areas. Particles with diameters ranging from 2.1–4.7 μm carried the most airborne bacteria. Therefore, particles with those dimensions should be the focus of future experimental research on occupational exposure, air quality improvement, and airborne transmission.	[55]
	Clinically Relevant Escherichia coli Isolates from Process Waters and Wastewater of Poultry and Pig Slaughterhouses in Germany	Germany	Poultry (n = 2) and pig (n = 2) slaughterhouses	Environmental health and food safety	Bacteria	Water samples from poultry (n = 82) and pigs (n = 67)	Passive methods (material collection)	Culture-based methods Antimicrobial susceptibility Molecular tools (whole-genome sequencing)	Selected *E. coli* isolates (n = 71) constituted a reservoir for 53 different antimicrobial resistance determinants and were assigned various sequence types, including high-risk clones involved in human infections worldwide. Through cross-contamination, these multidrug-resistant *E. coli* pathotypes may be introduced into the food chain. Moreover, inadequate wastewater treatment may contribute to bacterial dissemination into surface waters.	[29]
	The Interplay between Campylobacter and the Caecal Microbial Community of Commercial Broiler Chickens over Time	Italy	Poultry farms (n = 4)	Food safety	Bacteria	Cecal swabs (n = 320)	Passive methods (swabs)	Culture-based methods Molecular tools (RT-PCR/amplicon PCR)	Two out of four farms showed *Campylobacter* infection at different time points. Moreover, *Campylobacter* colonization dramatically influenced the microbiota richness, although to a different extent depending on the infection timing. Briefly, the evidence obtained in this study can be used to identify options to minimize the incidence of infection in primary production based on the targeted influence of birds’ intestinal microbiota, in order to reduce the risk of human exposure to *Campylobacter* by chicken meat consumption.	[43]
	Environmental Influences of High-Density Agricultural Animal Operation on Human Forearm Skin Microflora	USA	Animal farms (dairy and integrated farms: cattle, chicken, pig, sheep; n = 20)	Occupational health	Bacteria	Skin swabs from farm workers (n = 20)	Passive methods (swabs)	Molecular tools (16s rRNA gene sequencing)	Different microbial compositional patterns were found on skin of workers of different animal commodities. The alterations of forearm skin microflora in farm workers, influenced by their frequent farm animal operations, may increase their risk of skin infections with unusual pathogens and epidermal diseases.	[67]
	Occurrence of extended-spectrum betalactamase- producing Enterobacteriaceae, microbial loads, and endotoxin levels in dust from laying hen houses in Egypt	Egypt	Poultry farms (n = 28)	Occupational health and food safety	Bacteria Fungi Endotoxins	Settled dust from elevated surfaces inside the barn (n = 10), including the drinking system line, feeding system line, and ventilation opening	Passive methods (dust collection)	Culture-based methods Antimicrobial susceptibility MALDI-TOF (bacterial identification) LAL	Dust in Egyptian laying hen houses contains high concentrations of microorganisms and endotoxins, which might impair the health of birds and farmers when inhaled. Furthermore, laying hens in Egypt seem to be a reservoir for beta-lactamase (ESBL)-producing Enterobacteriaceae. Overall, farmers are at risk of exposure to ESBL-producing bacteria, and colonized hens might transmit these bacteria to the food chain.	[72]
	An observational field study of the cloacal microbiota in adult laying hens with and without access to an outdoor range	Netherlands	Poultry farms (n = 8)	Animal health	Bacteria	Cecal swabs (n = 100)	Passive methods (swabs)	Culture-based methods Molecular tools (16s rRNA gene sequencing)	Bacterial diversity was higher in Indoor layers than in outdoor layers, and indoor layers also had more variation in their bacterial community composition. No phyla or genera were found to be differentially abundant between indoor and outdoor poultry houses. The poultry house, farm, and rearing flock play a much greater role in determining the cloacal microbiota composition of adult laying hens.	[44]
	Dust at Various Workplaces—Microbiological and Toxicological Threats	Poland	Several workplaces (n = 4)(cement plant, composting plant, poultry farm, and cultivated area)	Occupational health	Bacteria Fungi	Air (n = 1) and settled dust (n = 1)	Active method (Air: DustTrak™ DRX Aerosol Monitor 8533 portable laser photometer, TSI) Passive methods (dust collection)	Culture-based methods Molecular tools (PCR) Cytotoxicity assay (A-549 MTT test)	Settled dust samples evidence the presence of 139 bacterial genera belonging to 8 classes and 107 fungal genera from 21 classes. In all tested settled dust samples, potentially allergenic molds were present, including *Aspergillus* sp. and *Penicillium* sp. (cement and composting plants) and *Cladosporium* sp. (cement plants and poultry farms)	[25]
	Hatchery workers’ IgG antibody profiles to airborne bacteriaPaul	Germany	Animal farms (duck hatchery) (n = 11)	Occupational health	Bacteria	Air samples Human sera (n = 10 workers	Active methods (filtration device using gelatin filters and polycarbonate filter, flow rate = 1.8 m^3^/h) Biological samples	Molecular tools (pulsed-field gel electrophoresis (PFGE); multiplex PCR and blaOXA-51-like and 16s rRNA gene sequencing) Fluorescence quantification	Despite long-term bioaerosol exposure, hatchery workers’ IgG antibody profiles to tested antigens did not differ substantially from those of the control group. However, increased workers’ titers to *Acinetobacter baumannii* and clinical relevance of this species should lead to further investigations regarding potential involvement in pathogenesis of occupational respiratory disorders.	[22]
	Epizootiological characteristics of viable bacteria and fungi in indoor air from porcine, chicken, or bovine husbandry confinement buildings	Korea	Animal farms (Swine, n = 5; chicken, n = 12; and cattle farms, n = 5)	Occupational and animal health	Bacteria Fungi Endotoxins	Air samples	Active methods (cascade impactor, flow rate = 28.3 ç/min, 20 min; PVC membrane filters (SKC) with 37 mm cassettes, flow rate = 2.0 L/min for 8 h, endotoxins)	Culture-based methods LAL	In chicken farms, a total of 22 Gram-positive bacterial species, three Gram-negative bacterial species, and five fungal species were identified. All broiler farms exceeded the recommended stocking density (0.066 m2/head), which may have led to the higher endotoxin concentrations in indoor dust from chicken farms than pig or cattle farms. Monitoring the indoor airborne endotoxin level was also found to be critical for risk assessment of health for animals or workers.	[61]
	Eggshells as a source for occupational exposure to airborne bacteria in hatcheries	Germany	Animal farms (turkey hatchery)	Occupational health	Bacteria	Air samples Turkey eggshell (n = 4)	Active methods (filtration devices (MD8 aluminum stacks, Sartorius, Göttingen, Germany) Bioaerosols were collected on gelatin filters (Ø 78 mm, 3.0 μm pore size (flow rate = 1.8 m^3^/h) Passive methods (material collection)	Culture-based methods Fluorescence quantification Molecular tools (16s rRNA gene sequencing)	*Enterococcus gallinarum* was found as the predominant species on turkey eggshells, both have been classified as risk group 2 microorganisms. During different work activities with poult and eggshell handling, concentrations of airborne Enterococci up to 1.3×104 cfu/m^3^ were found. After hatching of turkey poults, hatcher incubators and eggshell fragments provide appropriate conditions for excessive bacterial growth. Thus, high bacterial loads on eggshell fragments are a source of potentially harmful bioaerosols caused by air flows, poult activity, and handling of equipment.	[21]
	Evaluation of Microbiological and Chemical Contaminants in Poultry Farms	Poland	Poultry farms (n = 13)	Occupational and animal health	Bacteria	Air samples (n = 5) Settled dust (n = 3)	Active methods (aspirator (EAS 1203; Emio, Wrocław, Poland) DustTrak™ DRX aerosol monitor) Passive methods (dust collection)	Culture-based methods Cytotoxicity assay (A-549 MTT test) Chemical assessment (gas chromatographic and spectrophotometric methods (LC-MS/MS: secondary metabolites; GC/MS)	The airborne total dust concentration at poultry farms averaged 1.44 mg/m^3^ with a high percentage of the PM10 fraction. Microorganism concentrations in the settled dust were: 3.2 × 10^9^ cfu/g for bacteria and 1.2 × 10^6^ cfu/g for fungi. Potential pathogens (*Enterococcus* spp., *Escherichia coli*, *Salmonella* spp., *Aspergillus fumigatus*, *Paecilomyces variotii*) were also found. In conclusion, settled dust can be a carrier of microorganisms, odors, and secondary metabolites in poultry farms, which can be harmful to workers’ health.	[35]
	Detection of Airborne Bacteria in a Duck Production Facility with Two Different Personal Air Sampling Devices for an Exposure Assessment	Germany	Poultry farm (n = 2)	Occupational health	Bacteria	Air samples (n = 6)	Active methods (PGP filtration device with polycarbonate filters, pore size: 0.8 μm, Ø 37 mm; two-stage bioaerosol Cyclone 251, flow rate = 3.5 L/min)	Fluorescence quantification Molecular tools (restriction fragment length polymorphism (RFLP) analysis; 16s rRNA gene sequencing)	Detailed 16S rRNA gene sequence analyses showed potential exposure to risk group 2 bacteria at the hatchery workplace. A size fractionated sampling device revealed that pathogenic bacteria would reach the inhalable, thorax, and possibly alveolar fraction of lungs.	[27]
Scopus	Detection of Airborne Bacteria in a German Turkey House by Cultivation-Based and Molecular Methods	Germany	Poultry farms (n = 2)	Occupational health	Bacteria	Air samples (n = 2)	Active methods (filtration devices (MD8 aluminum stacks; Sartorius, Göttingen, Germany, through polycarbonate membrane filters, 0.80 lm pore size, flow rate = 28.1 L/min; all-glass impingers, AGI- 30; Ace Glass Inc., flow rate = 11.41 L/min)	Culture-based methods Molecular tools (PCR; 16s rRNA gene sequencing)	Microbial species with a potential health risk for employees (*Acinetobacter johnsonii, Aerococcus viridans, Pantoea agglomerans, and Shigella flexneri)* were identified. The animals seem to be the most important source of airborne microorganisms in the investigated turkey houses.	[23]
Scopus	Characterization of bacterial contaminants in the air of a duck hatchery by cultivation based and molecular methods	Germany	Poultry farm	Occupational health	Bacteria	Air samples (n = 10)	Active methods (filtration devices using polycarbonate filters (Ø 76 mm, 0.8 mm pore size) and gelatin filters (Ø 78 mm, 3.0 mm pore size, flow rate = 1.8 L/min)), MD8 aluminum stacks; Sartorius, Germany.	Culture-based methods Molecular tools (16s rRNA gene sequencing)	More than 50% of bacterial isolates were phylogenetically most closely related to bacterial species of risk group 2. There were high concentrations of risk group 2 bacteria, which have been implicated in different human respiratory disorders. Adequate breathing protection for employees is recommended during sorting of ducklings.	[28]
	Monitoring airborne biotic contaminants in the indoor environment of pig and poultry confinement buildings	USA	Animal farms (poultry and swine farm)	Occupational health	Bacteria	Air samples (n = 48)	Active methods (isokinetic sampling nozzle assembled with a polycarbonate cassette that housed a sterilized 37 mm glass fiber filter)	Molecular tools (16s rRNA pyrose sequencing; tetracycline resistance genes)	Bioaerosols in the confinement buildings were sporadically associated with genera of potential pathogens. Bacterial lineages present in the poultry bioaerosols clustered apart from those present in the pig bioaerosols. The abundance of different classes of tetracycline resistance genes also differed among the different animal confinement buildings.	[64]
	On-Site Investigation of Airborne Bacteria and Fungi According to Type of Poultry Houses in South Korea	Korea	Poultry farms (caged layer house n = 9; broiler house n = 9; layer house with manure belt n = 9)	Occupational health	Bacteria Fungi	Air samples (n = 5)	Active methods (one-stage viable particulate cascade impactor, Model 10–800, Andersen Inc., Bayport, MN, USA, flow rate of 28.3 L/min)	Culture-based methods	Among poultry buildings, the broiler house showed the highest exposure level and emission rate of total airborne bacteria and fungi, followed by the layer house with manure belt and the caged layer house. The highest exposure level and emission rate of airborne microorganisms found in the broiler house could be attributed to sawdust, which can be dispersed into the air by the movement of the poultry when it is utilized as bedding material.	[60]
	Exposure to Airborne Culturable Microorganisms and Endotoxin in Two Italian Poultry Slaughterhouses	Italy	Poultry slaughterhouses (n = 2)	Occupational health	Bacteria Fungi Endotoxins	Air samples (Poultry A n = 273; Poultry B n = 210) Workers’ personal air samples (Poultry A n = 5; Poultry B n = 2)	Active methods Portable air microbiological sampler -SAS Super ISO, PBI International, Milan, Italy, flow rate=100 L/min; Personal sampling pumps - Model Number SKC 224-PCXR8, Eighty Four, Pa., equipped with Button Aerosol Sampler and gelatin filters -GEL SKC, Inc., Pa, flow rate= 4 L/min;	Culture-based methods LAL	The microbial flora was dominated by Gram-negative and coagulase-negative *staphylococci* for bacteria and by species belonging to *Cladosporium, Penicillium*, and *Aspergillus* genera for molds. Overall, microbial levels were below the occupational limits. However, the microorganisms identified may exert adverse effects on exposed workers, in particular for those engaged in the early slaughtering stages, as evidenced by the presence of pathogenic species. Additionally, the detection of pathogenic bacteria near air handling units may constitute a risk to public health and of environmental pollution.	[42]
	Spread of airborne antibiotic resistance from animal farms to the environment: Dispersal pattern and exposure risk	China	Animal farms (poultry and dairy farm)	Human health due to environmental impact	Bacteria	Air samples (n = 4) Dust and animal feces samples	Active methods (portable high-volume sampler, HighBioTrap, Beijing dBlue Tech, Inc., Beijing, China, flow rate = 1000 L/min) Passive methods (material collection)	Culture-based methods Antimicrobial susceptibility Molecular tools (ABI QuantStudio™ 7 Flex RT-PCR; 16s rRNA gene sequencing)	Antibiotic resistance genes (ARGs) from bacteria were detected from upwind (50 m/100 m) and downwind (50 m/100 m/150 m) air environments, wherein at least 30% of bacterial taxa dispersed from the animal houses. Clinically important pathogens were identified in airborne culturable bacteria. *Staphylococcus*, *Sphingomonas*, and *Acinetobacter* genera were potential bacterial hosts of airborne ARGs. Airborne *Staphylococcus* spp. were isolated from the environment of the chicken farm (n = 148) and dairy farm (n = 87).	[53]
	Levels of bacterial endotoxin in air of animal houses determined with the use of gas chromatography—mass spectrometry and *Limulus* test	Poland	Animal farms (cow barns n = 4; piggeries n = 4; sheep sheds n = 4; poultry houses n = 4; horse stables n = 6) Buildings for storage of hay (n = 3)	Human health due to environmental impact and animal health	Endotoxins	Air samples (n = 2)	Active methods (portable single-unit aspirator AP-2A (TWO-MET, Zgierz, Poland) on pre-weighed glass fiber filters of diameter 37 mm and pore size 1.0 μm, flow rate = 2 L/min)	LAL Spectrophotometric methods (gas chromatography–tandem mass spectrometry (GC-MSMS)	The concentrations of airborne endotoxin determined with LAL test and GC-MSMS analysis exceeded the limits in most of the animal houses examined. Endotoxin in the concentrations detected in this study may present a respiratory hazard to both humans and livestock animals.	[34]
	Microbiological and chemical properties of litter from different chicken types and production systems	Lebanon	Poultry farms (n = 12)	Human health due to environmental impact and environmental health	Bacteria	Litter samples (n = 24)	Passive methods (material collection)	Culture-based methods Chemical analysis	*Staphylococcus* species were observed in the litter from free-range layers (*p* = 0.0077). *Staphylococcus* species in the litter as well as cadmium concentrations seem to be the most critical parameters presenting risks to the environment and human health.	[62]
	Quantifying Transmission of *Campylobacter jejuni* in Commercial Broiler Flocks	Australia	Poultry farms (n = 42)	Animal health	Bacteria	Surface swabs (fecal or cecal droppings, n = 10)	Passive methods (material collection)	Culture-based methods	The transmission rate estimate was 2.37 − 0.295 infections per infectious bird per day. Based on these results, colonized flocks consisting of 20,000 broilers would have an increase in within-flock prevalence to 95% within 4.4 to 7.2 days after colonization of the first broiler. Thus, interventions aimed at prevention of introduction and subsequent colonization by *Campylobacter* might be better targeted at the second half of the rearing period, which is considered a high-risk period.	[76]
	Presence and characterization of *Campylobacter jejuni* in organically raised chickens in Quebec	Canada	Poultry farms (n = 6)	Food safety	Bacteria	Cecal swabs (n = 30) Fecal matter (n = 30 g) Animal carcasses (birds, n = 10)	Passive methods (swabs, material collection)	Culture-based methods Antimicrobial susceptibility Molecular tools (PCR)	*Campylobacter jejuni* isolates were resistant to tetracycline, erythromycin, azithromycin, and clindamycin. Some organic chicken lots sampled in Quebec were positive for *C. jejuni*, which establishes this presence for the first time and suggests a possible contribution of these types of production to human campylobacteriosis.	[71]
	Risk characterization of antimicrobial resistance of Salmonella in meat products	Spain	Animal farms (poultry, pork, and beef farms; 95% industry and 5% retail)	Food safety	Bacteria	Animal carcasses (fresh poultry, n = 234); pork, n = 196); beef, n = 29; minced poultry, n = 151; pork, n = 1270; and beef, n = 170)	Passive methods (material collection)	Culture-based methods Antimicrobial susceptibility	*Salmonella* isolates found in poultry had a high level of resistance to nalidixic acid, while those found in pork were more resistant to tetracycline and ampicillin. Furthermore, 41% of *Salmonella* isolates were resistant to three or more antibiotics. Additionally, risk characterization was estimated. As a result, three cases were classified as “very high additional risk,” all of them in minced meat, two cases in poultry (gentamicin and nalidixic acid), and one in pork (ampicillin).	[45]
	Characterization of Antibiotic Resistance in *Enterobacteriaceae* From Agricultural Manure and Soil in Portugal	Portugal	Animal farms (poultry, n = 6) and dairy farms, n = 6)	Environmental health	Bacteria	Manure samples (n = 18) Soil samples (n = up to 15 cm)	Passive methods (material collection)	Culture-based methods Antimicrobial susceptibility Molecular tools (ARGs)	High multidrug resistance rates (>70%) were observed in both manure and soil samples. This resistance was higher in the poultry samples. Manured-soil isolates were more resistant to cefoxitin (91.7%), ulfamethoxazole/trimethoprim (79.2%), chloramphenicol (79.2%), and, to a lesser extent, tetracycline (12.5%). In short, the results obtained are important for soil management regarding resistance determinants spread through agricultural practices.	[37]
	Levels of bacterial endotoxin in the samples of settled dust collected in animal houses	Poland	Animal farms(poultry n = 4; sheep sheds n = 4; horse stables n = 6)	Occupational and animal health	Endotoxins	Settled dust samples (n = 14)	Passive methods (material collection)	LAL (endotoxins) Spectrophotometric methods (GC-MSMS)	The median concentrations of the endotoxin in dust determined with LAL tests in sheep sheds, poultry houses, and horse stables were 15,687.5 μg/g, 8081.8 μg/g, and 79.3 μg/g, respectively, while those determined with the GC-MSMS technique were 868.0 μg/g, 580.0 μg/g, and 496.0 μg/g, respectively. In conclusion, endotoxin in the concentrations detected in this study may present a respiratory hazard to both livestock animals and farm workers.	[33]
	Characterization of beta-lactamase and biofilm producing *Enterobacteriaceae* isolated from organized and backyard farm ducks	India	Animal farms (farm ducks, n = 8)	Human health due to environmental impact	Bacteria	Cloacal swabs (n = 202)	Passive methods (swabs)	Culture-based methods Antimicrobial susceptibility Molecular tools (PCR)	From 202 cloacal swabs of apparently healthy ducks, 109 (53–96%), 13 (6–44%), and 30 (14–85%) isolates were confirmed as *E. coli, Salmonella*, and *Klebsiella pneumoniae*, respectively. Most of the beta-lactamase and biofilm-producing *Enterobactriaceae* isolates exhibited phenotypical resistance against ampicillin, ampicillin/cloxacillin, and ceftriaxone. This study evidenced the ducks as a reservoir of beta-lactamase and biofilm-producing *Enterobactriaceae* which might enter the food chain to cause major public health hazards.	[77]
	More diversified antibiotic resistance genes in chickens and workers of the live poultry markets	China	Poultry farms (n = 21) and live poultry markets (LPMs) (n = 22)	Human health due to environmental impact	Bacteria	Bird fecal samples (n = 1215) Human fecal samples (n = 36) Material collection in 4 LPM environmental samples (soils, sediment, wastewater, and chopping boards, n = 4).	Passive methods (material collection)	Molecular tools (metagenomic sequencing (ARGs))	Some mobile ARGs, such as mcr-1 and tet(X3), identified in chicken farm LPMs, LPM workers, and LPM environments, were also harbored by human clinical samples. Resistomes were significantly different between the LPM workers and those who have no contact with the LPMs, and more diversified ARGs (188 types) were observed in the LPM workers. It is also worth noting that mcr-10 was identified in both human (5.2%, 96/1859) and chicken (1.5%, 14/910) gut microbiomes. These findings highlight the live poultry trade as an ARG disseminator into LPMs.	[56]
	Bacterial diversity characterization of bioaerosols from cage-housed and floor-housed poultry operations	Canada	Poultry farms (n = 30)	Occupational health	Bacteria and endotoxins	Air samples from cage-housed (CH, n = 15) and floor-housed (FH, n = 15) poultry operations	Active methods (Marple cascade impactor with weighed radial slit polyvinyl chloride (PVC) filters connected to a constant air flow pump—Universal 224-PCXR4; SKC, Eighty Four, PA, USA, six stages were included, flow rate = 2 L/min, over 4 hs)	Molecular tools (PCR; denaturing gradient gel electrophoresis (DGGE)) LAL Spectrophotometric methods (GC-MSMS)	Dust, endotoxin, and bacteria were significantly higher in personal bioaerosols of FH poultry operations than CH bioaerosols. Personal CH bioaerosols have a greater prevalence of bacteria, some of which have been shown to cause respiratory dysfunction. Therefore, bacterial diversity may help to explain the greater prevalence of respiratory symptoms in workers from CH operations.	[70]
	Characterization of drug-resistant Staphylococcus aureus isolated from poultry processing plants in Western Australia	Australia	Poultry processing plants (n = 2)	Food safety	Bacteria	Samples from broiler chickens and turkeys (n = 104) during the processing Samples from defeathering machinery and bleed drains (n = 22)	UK	Culture-based methods Antimicrobial susceptibility Molecular tools	One hundred and twenty-six *Staphylococcus aureus* were isolated from two poultry processing plants in Western Australia. Antimicrobial-resistant *S. aureus* were recovered from live incoming birds, equipment, and processed carcasses in the two processing plants. Indeed, forty-six (36.5%) of the isolates were resistant to six or more of the antimicrobial agents tested.	[75]
	Vancomycin-Resistant Enterococci (VRE) in Broiler Flocks 5 Years after the Avoparcin Ban	Denmark	Poultry farms where avoparcin had previously been used (n = 31) Poultry farms without avoparcin (n = 12)	Food safety	Bacteria	Cloacal swabs (n = 10)	Passive methods (swabs)	Culture-based methods Antimicrobial susceptibility Molecular tools (vanA PCR)	VRE were isolated from 104 of 140 (74.3%) broiler flocks reared in broiler houses previously exposed to avoparcin on conventional and extensive indoor broiler farms. Results demonstrated the extensive occurrence of VRE in broiler flocks even 5 years after the avoparcin ban in Denmark. The extensive occurrence of VRE in broiler flocks reported in this study indicates that consumers may still be exposed to VRE from poultry products despite the avoparcin ban.	[49]
	Personal Exposure to Airborne Dust and Microorganisms in Agricultural Environments	USA	Animal farms (swine, poultry, and dairy, n = 3) and grain farms (n = 3)	Occupational health	Bacteria Fungi	Air samples (swine n = 5; poultry n = 2; dairy n = 5; corn harvesting n = 6; soybean n = 3)	Active methods (prototype personal sampling, consists of seven components in each of the two sampling lines: sampling probe Tygon tubing, adaptor, metal sampling chamber, optical particle counter, 25 mm filter cassette and pump, flow rate = 10 L/min)	Culture-based methods Antimicrobial susceptibility Molecular tools (vanA PCR)	A large fraction (up to 37%) of particles from 2–10 μm was found to be fungal spores. Each type of agricultural environment was found to have specific characteristics of exposure. Harvesting was dominated by exposure to large dust particles with a large fraction of fungal spores, whereas the particle size distributions in animal confinements were dominated by small particles.	[65]
	Farm dust resistomes and bacterial microbiomes in European poultry and pig farms	European countries (Belgium, Bulgaria, Denmark, France, Germany, Italy, the Netherlands, Poland, and Spain)	Animal farms (poultry n = 12; swine farms n = 19)	Occupational health	Bacteria	Dust collection by electrostatic dust collector (n = 3) Fecal samples from poultry (n = 35) and workers (n = 44)	Passive methods (material collection)	Molecular tools (metagenomic sequencing (ARGs))	The farm dust resistome contained a large variety of ARGs; more than the animal fecal resistome. The farm dust resistome from European poultry and pig farms is equally or more abundant and rich than the resistome of poultry and pig feces and farmers. A positive association between on-farm antimicrobial usage in animals on the farm and the total abundance of the dust resistome was found. Briefly, poultry and pig farm dust resistomes are rich and abundant and associated with the fecal resistome of the animals and the dust bacterial microbiome	[51]
	Fluoroquinolone-resistant *Escherichia coli* isolated from healthy broiler s with previous exposure to fluoroquinolones: Is there a link?	Iran	Poultry farms (n = 7)	Human health due to environmental impact	Bacteria	Samples from broiler chickens and turkeys previously exposed to both quinolone (flumequine) and fluoroquinolone (n = 95)	UK	Culture-based methods Antimicrobial susceptibility	The differences between ciprofloxacin resistance rates in strains from chickens with previous exposure to fluoroquinolones compared with isolates from chickens without a history of drug use were significant (49.5% vs. 33.7%, p =/0.0461). It seems that use of fluoroquinolones constitutes a major selective pressure for resistance.	[63]
	Enumeration of *Campylobacter* spp. in Broiler Feces and in Corresponding Processed Carcasses	EUA	Poultry farms (n = 20)	Food safety	Bacteria	Bird fecal samples (n = 50) Bird carcasses before they entered the chill tank (n = 50) and after being fully processed (n = 50)	Passive methods (material collection)	Culture-based methods Antimicrobial susceptibility	Individual birds within each of the flocks involved in the current study were 70 to 100% colonized prior to loading and transport. Levels of *Campylobacter* spp. found in production and in processing were not strongly correlative, indicating the existence of complex parameters involving production factors and variables associated with flock transport and the processing of the broilers. The sources of *Campylobacter* sp. appear to be diverse, and discussion regarding the optimum approach for the control of the organism during poultry production remains lively.	[69]
	A prospective Study of Management and Litter Variables Associated with Cellulitis in California Broiler Flocks	USA	Poultry farms (n = 5)	Animal health	Bacteria	Litter samples (n = 3, 60 g)	Passive methods (material collection)	Culture-based methods	There was a positive association between the quantity of Gram-negative bacteria in the litter in the front third of the house (the brooding area) during the brooding period and the percentage of cellulitis.	[68]
	Fungal aerosol in the process of poultry breeding quantitative and qualitative analysis	Poland	Poultry farms (n = 5)	Occupational health	Fungi	Air samples (n = 11)	Active methods (filtration method, GilAir 5 pump—Sensidyne, Clearwater, Florida, USA; open-faced aerosol sampler Two-Met, Zgierz, Poland, with a GF/A filter, Whatman International Ltd., Maidstone, Kent, UK, of a 37 mm diameter, flow rate = 2 L/min)	Culture-based methods	In 45% of the taken samples, airborne mesophilic fungal levels exceeded the reference value recommended in Poland for occupational environment exposure. Briefly, facilities of poultry farms are contaminated with high concentrations of fungal aerosols, especially in the colder season. Additionally, potential pathogenic microorganisms were present, which may pose a risk to farm workers’ health.	[36]
	Seasonal biodiversity of pathogenic fungi in farming air area. Case study.	Poland	Poultry farm	Human health due to environmental impact	Fungi	Air samples (indoor n = 4, outdoor n = 4)	Active methods (impaction method, Merck MAS-100, flow rate = 100 L/min)	Culture-based methods	The most common airborne fungi, inside the poultry house, as well as in the surrounding areas, were *Penicilium* sp., *Aspergillus* sp., *Cladosporium* sp., and *Alternaria* sp. The majority of the identified fungal species were characterized as potential allergens and exposure to their spores may provoke immune response in susceptible individuals.	[32]94b
Web of Science	Aerosol Concentrations and Fungal Communities Within Broiler Houses in Different Broiler Growth Stages in Summer	China	Poultry farms (n = 3)	Human health due to environmental impact and animal health	Fungi	Air samples (n = 3)	Active methods (Andersen six-stage sampler ZR-2001, Zhongrui, Qingdao, China, flow rate = 28.3 L/min, 2 min; biosampler (ZR- 2000, Zhongrui, Qingdao, China, flow rate = 5–35 L/min))	Molecular tools (PCR)	The concentration of fungal aerosols in the poultry houses increased as the ages of the broilers increased, which was also accompanied by gradual increases in the variety and diversity indices of the fungal communities in the air of the poultry houses. Overall, the dominant fungal genera found may be harmful to the health of poultry and human beings. Thus, permanent monitoring of microbial air quality in chicken houses is necessary.	[78]
	Respiratory health disorders associated with occupational exposure to bioaerosols among workers in poultry breeding farms	Egypt	Poultry farms (n = 10)	Occupational health	Bacteria Fungi	Air samples (n = 10) Swabs (workers’ nose and throat, n = 56)	Active methods (Andersen six-stage impactor, Model 10–710, Andersen Instruments, Atlanta, GA, USA, flow rate = 28.3 L/min, 0.5 to 2 min; spirometer, MEE Spiro PFT touch, Germany) Passive methods (swabs)	Culture-based methods Questionnaire Spirometric measures	The percentage of total positive cultured (bacterial and fungal) was 35.7% among the poultry breeding farm workers. About one third of the studied farm workers (30.4%) were a carrier for *S. aureus* in the nose and throat compared with 12.5% of the control group. Additionally, *Aspergillus* species were present in air samples as well as in human samples. These results suggest that poultry breeding farms might be vehicles of human fungal infections.	[74]
	Bacterial communities in PM2.5 and PM10 in broiler houses at different broiler growth stages in spring	China	Poultry farms (n = 3)	Animal health	Bacteria	Air samples (n = 3)	Active methods (ZR-3920 environmental air particulate matter sampler using 9 cm Tissuquartz™ filters, Pall, Port Washington, NY, USA, flow rate = 100 L/min, 48 h)	Molecular tools (PCR; 16s rRNA gene sequencing	Results revealed that PM2.5, PM10 airborne microbes gradually increased during the broiler growth cycle in poultry houses. Additionally, some potential or opportunistic pathogens were found in the broiler houses at different growth stages	[57]
	Size-related bacterial diversity and tetracycline resistance gene abundance in the air of concentrated poultry feeding operations	China	Poultry farms (n = 8)	Occupational and animal health	Bacteria	Air samples (outside the office; inside/outside the layer house; inside/outside the broiler house n = 5)	Active methods (eight-stage non-viable Andersen samplers coupled with quartz fiber, flow rate = 28.3 L/min, 48 h)	Molecular tools (qPCR; 16s rRNA, tetL, tetW, and *E. coli* gene sequencing	The richness of biological genera in the urban atmospheric environment was lower than in concentrated poultry feeding operations. The bacterial lineages of bioaerosols present in the seven size stages for layers clustered apart from those for broilers, suggesting that the type of poultry house is a more important factor than the particle size in shaping the microbial communities. Results suggest that bioaerosols containing antibiotic resistance genes and potential airborne pathogens from animal feeding operations can be efficiently transferred to the nearby environment.	[54]
	Slaughterhouses Fungal Burden Assessment: A Contribution for the Pursuit of a Better Assessment Strategy	Portugal	Poultry (n = 1), swine/bovine (n = 1), and large animal slaughterhouses (n = 1)	Occupational health	Fungi	Air samples: (poultry n = 6; swine/bovine n = 6; large animal slaughterhouses n = 6) Surface samples (poultry floor n = 6; swine/bovine walls n = 6; animal floor slaughterhouses n = 6)	Active methods (impaction method, Millipore air tester, Millipore, flow rate = 140 L/min) Passive methods (swabs)	Culture-based methods Molecular tools (qPCR)	Poultry and swine/bovine slaughterhouses each presented two sampling sites that surpass the guideline of 150 CFU/m^3^. A. *fumigatus* complex was identified through molecular tools in six sampling sites. Results evidence indicators that are representative of harmful fungal contamination in these settings.	[39]
	Occupational exposure to airborne microorganisms, endotoxins and β-glucans in poultry houses at different stages of the production cycle	Poland	Poultry farms (n = 3)	Occupational health	Bacteria Fungi, endotoxins, and β-glucans	Air samples (different stages of chicken production cycle, n = 3)	Active methods (six-stage Andersen impactor Model 10–710, Andersen Instruments, Atlanta, GA, USA, flow rate = 28.3 L/min, 0.5 to 2 min; Harvard impactors with 37 mm Teflon filters with 1 μm pore size, SKC Ltd., measurements of PM10, flow rate = 10 L/min, 4 h; filter samplers, button aerosol sampler, SKC Ltd., Eighty Four, PA, USA, clipped onto a worker’s collar. Collected on gelatin filters of 25 mm with a pore size of 3 μm, SKC Ltd., flow rate = 4 L/min, 30 min; Harvard impactors with 37 mm Teflon filters with 1 μm pore size, SKC Ltd.,measurements of PM10, flow rate =10 L/min, 4 h)	Culture-based methods LAL Quantitative kinetic Glucatell assay (β-glucans)	The level of PM10 in poultry facilities did not exceed 4.5 mg/m^3^. After the flock entered the clean house, the level of endotoxins and β-glucans increased from below detection limit to 8364 ng/m^3^ and from 0.8 ng/m^3^ to 6886 ng/m^3^, respectively. The results show that professional activities in poultry farms are associated with constant exposure to bioaerosol, which may pose a health hazard to workers. In addition, it was found that workers’ exposure to airborne microorganisms increased with consecutive stages of the chicken production cycle.	[31]
	Fungal Contamination of Poultry Litter: A Public Health Problem	Portugal	Poultry farms (n = 7)	Occupational health	Fungi	Air samples (n = 27) Litter collection (fresh n = 7, aged n = 14; 10 gr)	Active methods (impaction method) Passive methods (material collection)	Culture-based methods Molecular tools (qPCR)	A significant positive correlation was found between litter fungal contamination (CFU/g) and air fungal contamination (CFU/m^3^). Spreading of poultry litter in agricultural fields is a potential public health concern, since keratinophilic (*Scopulariopsis* and *Fusarium* genera) as well as toxigenic fungi (*Aspergillus*, *Fusarium*, and *Penicillium* genera) were isolated.	[38]
	The variability of bacterial aerosol in poultry houses depending on selected factors	Poland	Poultry farms (n = 5)	Occupational health	Bacteria	Air samples (n = 11)	Active methods (filtration method using the GilAir 5 pump, Sensidyne, Clearwater, Florida, USA; open-faced aerosol sampler, Two-Met, Zgierz, Poland, with a GF/A glass microfiber filter, Whatman International Ltd., Maidstone, Kent, UK, with a pore size of 1.6 μm, flow rate = 2 L/min, 4–6 h)	Culture-based methods	The lowest concentrations of total bacteria were obtained in those buildings where one-day-old chickens were kept. It was shown that for most of the investigated livestock premises the total bacterial concentrations exceeded the reference value of 1.0 × 10^5^ cfu/m^3^. Furthermore, pathogenic microorganisms which are a potential threat to human health were found among the identified bacteria.	[30]
	Endotoxin concentration in poultry houses for laying hens kept in cages or in alternative housing systems	France	Poultry farms n = 21 (caged n = 8, free-cage n = 13)	Occupational health	Endotoxins	Air samples (n = 2) Personal air samples (n = 2)	Active methods (CAP 10, ARELCO, Auxerre, France, flow rate = 1 L/min, 7, 8 h; personal air samples collected in 37 mm diameter glass fiber filters with a pore size of 0–5 mm; Millipore AP4003705, St Quentin, France), aseptically placed in three-part polystyrene filter holders, Millipore M000037AO, in a constant airflow pump, SKC 224, PCTX8, ARELCO, flow rate = 1 L/min, 6 h)	LAL	The endotoxin concentrations in the ambient air, and to which workers were exposed, appeared to be high in comparison with the threshold of 50 EU/m^3^ over 8 h. Differences in dust and endotoxin concentrations between the cage and alternative systems may be due to the presence of litter and to the greater activity of the hens in the on-floor buildings. Effective methods to reduce worker exposure to air contaminants in laying houses still need to be developed.	[48]
	Culture-Independent Characterization of Bacteria and Fungi in a Poultry Bioaerosol Using Pyrosequencing: A New Approach	USA	Poultry farm	Occupational health	Bacteria Fungi	Air samples (n = 29)	Active methods (inhalable sampler, IOM, SKC Inc., Eighty Four, PA, was loaded with a 25 mm, sterile, gelatin membrane filter with a pore size of 3 μm, SKC Inc., connected to a personal sampling pump Model 210–5000, SKC Inc, flow rate = 2 L/min, 8 h)	Molecular tools (tag-encoded flexible (FLX) amplicon pyrosequencing (bTEFAP) and fungal tag-encoded flexible (FLX) amplicon pyrosequencing (fTEFAP))	Concerning bacteria and fungi detected, 116 and 39 genera were identified, respectively. Among bacteria, *Staphylococcus cohnii* was present in the highest proportion (23%). The total inhalable bacteria concentration was estimated to be 7503 cells/m^3^. Among the fungi identified, *Sagenomella sclerotialis* was present in the highest proportion (37%). *Aspergillus ochraceus* and *Penicillium janthinellum* were also present in high proportions. Briefly, a limited amount of information exists on the bioaerosols present in a poultry production environment. Future work should include an expanded sampling plan and additional production sites for enhanced generalizability of the results.	[66]
	Air biocontamination in a variety of agricultural industry environments in Egypt: a pilot study	Egypt	Several workplaces (poultry farm, flourmill, textile and food industry)	Occupational health	Bacteria Fungi	Air samples (poultry farm n = 4; flourmill n = 8; textile n = 8 and food industry n = 2)	Active methods (liquid impinger AGI-30, Vineland, New Jersey, USA, containing 20 mL phosphate buffer, KH2PO4 0.4%, K2HPO4 1.36%, flow rate = 12.5 L/min, 15 min). Gravimetric sampler: open-faced holder with cellulose nitrate membrane filters, pore size 0.45 lm, diameter 25 mm; Whatman, Maidstone, UK, flow rate = 8 L/min, 2 h)	Culture-based methods	The highest median indoor concentration of culturable airborne bacteria (6.23 × 10^5^ CFU/m^3^) was found at the occupied poultry farm. Meanwhile, the highest median indoor concentration of culturable airborne fungi (3.15 × 10^4^ CFU/m^3^) was found at the flourmill site. In short, workers in Egyptian agriculture-related industries are exposed to aerosolized particulate matter and microbial concentrations.	[73]
	Detection of *Jeotgalicoccus* spp. In poultry house air	Germany	Poultry farms (n = 3) (turkey, chicken, and duck houses), duck slaughterhouse (n = 3)	Occupational health	Bacteria	Air samples (turkey n = 9; duck n = 9; chicken n = 9 farms; duck slaughterhouses n = 9)	Active methods (filtration devices, MD8 aluminum stacks; Sartorius, Germany, with polycarbonate membrane filters, Isopore ATTP 0.8 lm pore size; Millipore, for poultry farms, flow rate = 27.2 L/min, 2 h; personal air samplers, PGP/GSP-3.5; BIA, Germany, in combination with specific SG-10 (GSA) pumps, for duck farms, with polycarbonate filters, 0.8 lm pore size; 3.7 cm; Whatman, flow rate= 3.5 L/min, 8 h)	Molecular tools (16s rRNA gene sequencing)	Estimated concentrations by quantitative real-time PCR analyses revealed cell numbers between 10^4^ and 10^6^ of *Jeotgalicoccus* sp. per m^−3^ of air in turkey, duck, and chicken houses. These results indicated the remarkable proportion (1–39%) of total cell counts and the hitherto unknown wide distribution of *Jeotgalicoccus* sp. in the poultry rearing industry.	[26]
	Direct Detection of *Salmonella* Cells in the Air of Livestock Stables by Real-Time PCR	Germany	Poultry farms (broiler farm n = 2, duck farm n = 1)	Occupational health	Bacteria	Air samples (turkey n = 9; duck n = 9; chicken n = 9 farms; duck slaughterhouses n = 9)	Active methods (filtration devices, MD8 aluminum stacks; Sartorius, Germany, with polycarbonate membrane filters, Isopore ATTP 0.8 lm pore size; Millipore, for broiler farms, flow rate = 27.2 L/min, 2 h; personal air samplers, PGP/GSP-3.5; BIA, Germany, in combination with specific SG-10 (GSA) pumps, for duck farm, on polycarbonate filters, 0.8 lm pore size; 3.7 cm; Whatman, flow rate = 3.5 L/min, 8 h)	Culture based-methods Molecular tools (qPCR)	The results demonstrate airborne *Salmonella* sp. workplace concentrations in poultry production of up to 3.3% of 49,6-diamidino-2-phenylindole-counted total cell numbers. The risk of infection at these working places seems quite high.	[24]
	A case study of airborne culturable microorganisms in a poultry slaughterhouse in Styria, Austria	Austria	Poultry slaughterhouse	Occupational health	Bacteria Fungi	Air samples (Hanging area and eviscerating area, n = 2)	Active methods (Andersen six-stage viable cascade impactor, ACFM, Graseby, USA, flow rate = 28.3 L/min, 15s; impingement method, SKC biosampler, SKC, USA, flow rate = 10 L/min, 10 min)	Culture based-methods	The median concentration of airborne mesophilic bacteria was 1.7 × 10^6^ CFU/m^3^ in the processing area of the “moving rail,” which is 8000 times higher than the background concentration of residential areas (approx. 210 CFU/m^3^). Results evidence that poultry slaughterhouse employees are exposed to high concentrations of airborne microorganisms throughout the entire work time without using a respiratory protective device.	[46]
	Exposure Levels of Airborne Bacteria and Fungi in Korean Swine and Poultry Sheds	Korea	Animal farms (poultry n = 4; swine farm n = 2)	Occupational health	Bacteria Fungi	Air samples (winter n = 68, summer n = 60)	Active methods (single-stage Andersen samplers with 400 0.25 mm holes, flow rate = 28.3 L/min, 0.5–2 min)	Culture based-methods	*Aspergillus, Cladosporium*, and *Penicillium* represented most of the fungi (96% and 82% in the swine sheds for winter and summer, respectively, and 69% in the poultry sheds). Many microbial concentrations exceeded the Korean indoor bioaerosol guideline of 800 CFU/m^3^.	[58]
	Airborne Fungi In Industrial Environments—Potential Agents Of Respiratory Diseases	Lithuania	Several workplaces (poultry farm, swinery, feed preparing and storing house, grain mill, wooden panel producing factory, and organic waste recycling facilities, n = 6)	Occupational health	Fungi	Air samples	Active methods (AGI-30 all glass impinger, Ace Glass Inc., Vineland, NJ, USA; cut-off 0.31 μm; air filtering through 47 mm cellulose membrane, Whatman plc, Kent, UK, pore size not specified, mounted on a plastic filter holder, flow rate = 0.001 m3/min, 15 min; Krotov 818 impactor was operated for 1 and 2 min at the flow rate = 0.025 m^3^/min, 1–2 min)	Culture based-methods	Thirty-one species attributed to thirteen fungal genera were isolated from the poultry house air. According to evidence, the majority of the identified fungal species found in industrial environments are characterized as allergenic and exposure to their spores may provoke adverse health effects in susceptible individuals.	[47]
	Air contaminants in different european farming environments	European countries (Denmark, Switzerland, Spain)	Animal farms (pig farm in Denmark, poultry farm in Switzerland, and greenhouse in Spain)	Occupational health	Bacteria Fungi Endotoxins	Air samples Personal air samples	Active methods (polycarbonate filters with a pore size of 0.4 μm and a diameter of 25 mm were placed on cellulose support pads and sealed in presterilized carbon-filled polypropylene air monitoring cassettes, Pegasus Labor, Duesseldorf, Germany, connected to portable battery-operated pumps, flow rate = 1 L/min; airborne dust (PM10) was collected on preweighed 37 mm diameter glass fiber filters fixed in threaded holders; personal air sampler, battery-operated pumps, flow rate = 3.5 L/min)	Culture based-methods Fluorescence quantification LAL	The highest total dust concentrations were found in poultry houses in Switzerland with median concentrations of 7.01 mg/m^3^. The highest total and active fungus concentrations were detected in poultry houses compared to pig houses and greenhouses. Additionally, bacterial concentrations were high in all animal houses. The exposure level found in this study might put the farmers at risk of respiratory diseases.	[52]
	Exposure assessment and lung function in pig and poultry farmers	Denmark and Switzerland	Animal farms (poultry farm, Denmark; Swine farm, Switzerland)	Occupational health	Bacteria Fungi Endotoxins	Air samples Personal air samples (poultry farmers n = 36, pig farmers n = 40)	Active methods (polycarbonate filters with a pore size of 0.4 μm and a diameter of 25 mm placed on cellulose support pads and sealed in presterilized carbon-filled polypropylene air monitoring cassettes, airflow = 1 L/min; dust was collected on preweighed 37 mm diameter glass fiber filters fixed in threaded holders, flow rate = 3.5 L/min)	Questionnaires Spirometry LAL Fluorescence quantification	Results evidence that factors related to work in the housing areas of pigs and poultry (variables of ventilation and feeding management) were significantly associated with decrements in lung function. Additionally, higher temperatures inside the pig houses were significantly negatively associated with lung function in pig farmers. Overall, lung function results were significantly associated with ventilation of the animal houses.	[50]

## Data Availability

No applicable.

## Supplementary Materials

The following supporting information can be downloaded at: https://www.mdpi.com/article/10.3390/toxics11040374/s1, Table S1: PRISMA checklist.
