# Supplementary material for: One Health Approach to Tackle Microbial Contamination on Poultries—A Systematic Review
Source: Toxics. 2023 Apr 14;11(4):374. doi: 10.3390/toxics11040374 (PMC10142658; doi:10.3390/toxics11040374)
Supplement: Supplementary file 1 [file toxics-11-00374-s001.zip › toxics-2290454-supplementary.pdf]

toxics

MDPI

Supplementary Materials

One Health Approach to Tackle Microbial Contamination on Poultries—A Systematic Review

Bianca Gomes [1,2,*], Marta Dias [2,3], Renata Cervantes [2,3], Pedro Pena [2,3], Joana Santos [4], Marta Vasconcelos Pinto [5,6] and Carla Viegas [2,3]

Table S1. PRISMA Checklist (Adopted from [14]).

Section/Topic	#	Checklist Item	Reported On Page #
**Title**			
Title	1	Identify the report as a systematic review, meta-analyses.	1
**Abstract**			
Structured Summary	2	Provide a structured summary including, as applicable: background; objectives; data sources; study eligibility criteria, participants, and interventions; study appraisal and synthesis methods; results; limitations; conclusions and implications of key findings; systematic review registration number.	1
**Introduction**			
Rationale	3	Describe the rationale for the review in the context of what is already known.	2 and 3
Objectives	4	Provide an explicit statement of questions being addressed with reference to participants, interventions, comparisons, outcomes, and study design (PICOS).	3
**Methods**			
Protocol and Registration	5	Indicate if a review protocol exists, if and where it can be accessed (e.g., Web address), and, if available, provide registration information including registration number.	3
Eligibility Criteria	6	Specify study characteristics (e.g., PICOS, length of follow-up) and report characteristics (e.g., years considered, language, publication status) used as criteria for eligibility, giving rationale.	3
Information Sources	7	Describe all information sources (e.g., databases with dates of coverage, contact with study authors to identify additional studies) in the search and date last searched.	3
Search	8	Present full electronic search strategy for at least one database, including any limits used, such that it could be repeated.	4
Study Selection	9	State the process for selecting studies (i.e., screening, eligibility, included in systematic review, and, if applicable, included in the meta-analysis).	4
Data Collection Process	10	Describe method of data extraction from reports (e.g., piloted forms, independently, in duplicate) and any processes for obtaining and confirming data from investigators.	4
Data Items	11	List and define all variables for which data were sought (e.g., PICOS, funding sources) and any assumptions and simplifications made.	4
Risk of Bias in Individual Studies	12	Describe methods used for assessing risk of bias of individual studies (including specification of whether this was done at the study or	4

		outcome level), and how this information is to be used in any data synthesis.	
Summary Measures	13	State the principal summary measures (e.g., risk ratio, difference in means).	4
Synthesis of Results	14	Describe the methods of handling data and combining results of studies, if done, including measures of consistency (e.g., I^2) for each meta-analysis.	4
Risk of Bias Across Studies	15	Specify any assessment of risk of bias that may affect the cumulative evidence (e.g., publication bias, selective reporting within studies).	4
Additional Analyses	16	Describe methods of additional analyses (e.g., sensitivity or subgroup analyses, meta-regression), if done, indicating which were pre-specified.	4
**Results**			
Study Selection	17	Give numbers of studies screened, assessed for eligibility, and included in the review, with reasons for exclusions at each stage, ideally with a flow diagram.	4, Figure 1
Study Characteristics	18	For each study, present characteristics for which data were extracted (e.g., study size, PICOS, follow-up period) and provide the citations.	6-34, Table 2
Risk of Bias within Studies	19	Present data on risk of bias of each study and, if available, any outcome level assessment (see item 12).	4
Results of Individual Studies	20	For all outcomes considered (benefits or harms), present, for each study: (a) simple summary data for each intervention group, (b) effect estimates and confidence intervals, ideally with a forest plot.	Table 2
Synthesis of Results	21	Present results of each meta-analysis done, including confidence intervals and measures of consistency.	Table 2
Risk of Bias Across Studies	22	Present results of any assessment of risk of bias across studies (see Item 15).	4
Additional Analysis	23	Give results of additional analyses, if done (e.g., sensitivity or subgroup analyses, meta-regression (see Item 16)).	Not applicable
**Discussion**			
Summary of Evidence	24	Summarize the main findings including the strength of evidence for each main outcome; consider their relevance to key groups (e.g., healthcare providers, users, and policy makers).	35-37
Limitations	25	Discuss limitations at study and outcome level (e.g., risk of bias), and at review level (e.g., incomplete retrieval of identified research, reporting bias).	38-39
Conclusions	26	Provide a general interpretation of the results in the context of other evidence and implications for future research.	39
**Funding**			
Funding	27	Describe sources of funding for the systematic review and other support (e.g., supply of data); role of funders for the systematic review.	39
